# In vitro comparison of flexural strength and elastic modulus of three provisional crown materials used in fixed prosthodontics

**DOI:** 10.4317/jced.51136

**Published:** 2013-12-01

**Authors:** Vachan Poonacha, Seema Poonacha, Basavaraj Salagundi, P L. Rupesh, Rohit Raghavan

**Affiliations:** 1 MDS, Senior Lecturer. Dept. of Prosthodontics, Coorg Institute of Dental Sciences, Virajpet, S-Coorg, Karnataka India; 2 MDS, Senior Lecturer. Dept. of Conservative Dentistry And Endodontics, Coorg Institute of Dental Sciences, Virajpet, S-Coorg, Karnataka India; 3 MDS, Professor- Dept. of Prosthodontics, Coorg Institute of Dental Sciences, Virajpet, S-Coorg, Karnataka, India; 4 MDS, Professor and Head. Dept. of Prosthodontics, Coorg Institute of Dental Sciences, Virajpet, S-Coorg, Karnataka, India

## Abstract

Objectives: To evaluate and compare the flexural strength and the elastic moduli of three provisional crown materials (methyl methacrylate based autopolymerized resin, bis acryl composite based autopolymerized resin and urethane dimethacrylate based light polymerized resin) after storing in artificial saliva and testing at intervals of 24 hours and 7 days. Study design: A metal master mould with four slots of dimensions 25x2x2 mm was fabricated to obtain samples of standard dimensions. A total of 135 specimens were thus obtained with 45 each of three provisional materials. Further 15 samples of each group were tested after storing for one hour at room temperature and again at intervals of 24 hours and 7 days after storing in artificial saliva. Three point flexural tests were carried out in the universal testing machine to calculate the flexural strength and the elastic modulus. The changes were calculated and data was analyzed with Fisher’s test and ANOVA. Results: The flexural strength of the methyl methacrylate resin reduced significantly while bis-acrylic composite resin showed a significant increase in its flexural strength after storing in artificial saliva for 24 hours and the values of both remained constant thereafter. Contrary to these findings, light polymerized resin showed a significant decrease in flexural strength after storing in artificial saliva for 24 hours and then significantly increased in flexural strength after 7 days. However the changes in the values for elastic modulus of respective materials were statistically insignificant. Conclusion: Methacrylate based autopolymerizing resin showed the highest flexural strength and elastic moduli after fabrication and after storing in artificial saliva and for 24 hours and 7 days. Bis-acrylic composite resin showed the least flexural strength and elastic moduli.

** Key words:**Provisional restorations, interim restorations, Methyl Methacrylate, composite restoration, flexural strength, elastic moduli.

## Introduction

Provisional crowns and fixed partial dentures (FPDs) are essential components of fixed prosthodontic treatment (1). Definitive crown and fixed partial denture (FPD) restorations are usually a multiple-dental-visit procedure which requires that the interim restoration mimic the planned final restoration independent of the restorative material(s) used for that restoration (2). The function of provisional restorations are varied and aims to cover exposed dentine to prevent sensitivity and plaque buildup, to prevent unwanted tooth movement, to maintain function adequately, to facilitate oral hygiene, prevent gingival overgrowth, to provide adequate interim appearance and to assess the effect of aesthetic and occlusal changes (3). In a given clinical circumstance when considerable masticatory forces are applied, fracture of the long span restoration is more likely than a short span (4) . Temporary materials have changed immensely since their early days in the 1930s- from acrylics and premade crown forms to newer bisacryl materials and computer-aided design/computer-aided manufacturing (CAD/CAM) generated restorations (5). Though extensive research has been done regarding the fracture resistance of various available provisional restorative materials there however is a paucity of information in the literature regarding the flexural strength and elastic moduli of provisional restorative materials in simulated in vivo conditions. Hence this study has embarked upon the investigation of the flexural strength and elastic modulus of three common provisional crown materials in simulated intra oral conditions.

The aim of this study was to evaluate and compare the flexural strength and elastic moduli of three provisional crown materials (methyl methacrylate based autopolymerized resin, bis acryl composite based autopolymerized resin and urethane dimethacrylate based light polymerized resin) at room temperature and study the change in flexural strength and elastic moduli of these three materials after storing in artificial saliva for 24 hours and 7 days.

## Material and Methods

**Figure 1:**
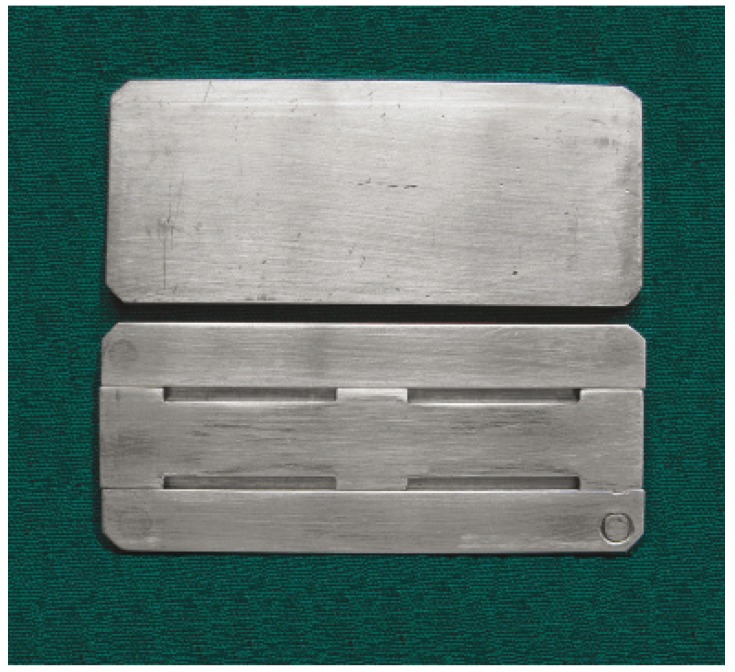
Metallic master mould.

The three provisional crown materials that were tested in this study were methyl methacrylate based autopoly-merized provisional crown material (DPI ™ Self – Cure Tooth Molding Powder, Dental Products of India, 9, Wallace treat, Mumbai), bis acrylic composite based autopolymerized provisional crown material (Protemp™ II – 3M ESPE AG Dental Products D-82229 Seefeld - Germany) and a urethane dimethacrylate based light polymerized provisional crown material (Revotek™ LC– GC DENTAL PROUCTS CORP, 2-285 TORI-MATSU-CHO, KASUGAI, AICHI, JAPAN). A metallic master mould was fabricated with three metallic plates. The plate in the center had four slots of dimensions 25x2x2 mm to which the materials under study could be filled to get samples of similar dimensions (Fig. 1). The provisional crown materials were mixed according to manufacturer’s instructions, injected into the metallic mould and held under compression. For light polymerizing material a glass cover plate was used to pack the material since it had to be light cured.

The composite based autopolymerized provisional crown material was supplied in the form of three pastes. Ratio of the pastes was according to the number of snap turns as recommended by the manufacturer. The resin was mixed for 10 seconds at room temperature; it was loaded into a syringe and injected into the mould which was well lubricated. Two minutes after the mixing had commenced, the sample was retrieved from the mould.

The light polymerized provisional crown material was supplied in a paste form which could be light polymerized. The material was packed in the mould and after the mould was filled with resin, a well lubricated glass plate was placed over it and was light cured for forty seconds with a light cure unit. The glass plate was then removed and the sample was retrieved.

The acrylic based autopolymerized provisional crown material was supplied in powder and liquid form. One minute and 50 seconds after the mixing had commenced, the sample was retrieved from the mould. Excess resin was removed from all the three types of samples using fine grit abrasive paper and dimensions were confirmed using an electronic vernier caliper.

**Figure 2:**
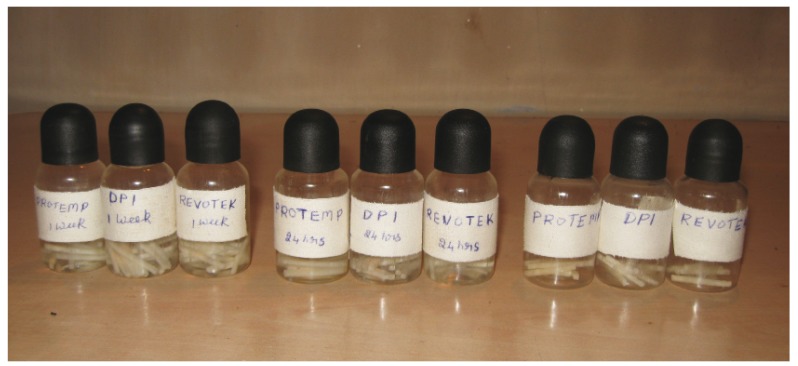
Specimens stored.

135 samples with dimensions of 25x2x2mm (American National Standards Institute/American Dental Associa-tion specification no. 27) (1) of all the three materials were prepared in a similar way. Out of these 15 samples each of the three different provisional crown materials was stored at room temperature for one hour under normal atmospheric conditions before testing. Another 15 samples each of the three provisional crown material was stored in artificial saliva (1 L double distilled H2O, 1.6802g NaHCO3, 0.41397g NaH2PO4•H2O, and 0.11099 g CaCl2) (1,6) for 24 hours at room temperature under normal atmospheric conditions before testing. A further set of 15 samples each of the three provisional crown materials were stored in artificial saliva for 7 days at room temperature under normal atmospheric conditions before testing (Fig. 2).

**Figure 3:**
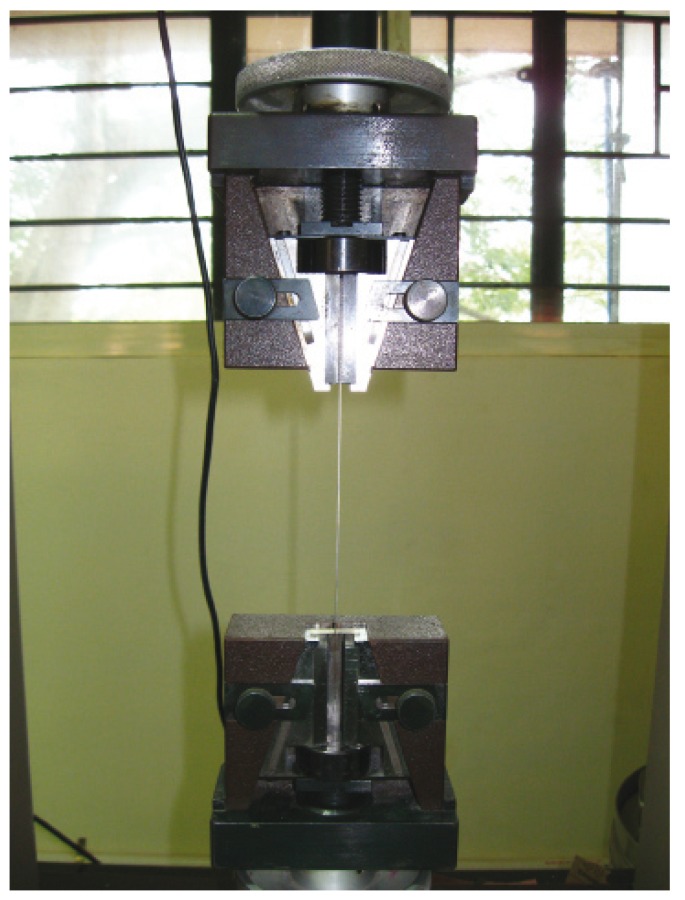
Sample being tested.

Three point flexural tests were carried out for all the samples in the universal testing machine (Fig. 3). The fracture load i.e., load at which the specimen fractured and the deviation was noted on the specific meters. The flexural strength (S) and the elastic modulus (E) were calculated using the following formula:

S = 3Pl / 2wt2, E = Pl3 / 4wt3?

Where P-Applied load (N), l-Span (m), w- Width (m), t-Thickness (m), and ?-Deflection (m).

Data were compared with analyses of variance and Fisher’s least significant difference tests (? = 0.05). Two-way analyses of variance (ANOVA) (? = 0.05) was done to determine if the difference in the values between the groups were statistically significant.

## Results

The study evaluated the flexural strength and elastic moduli of three provisional crown materials (methyl methacrylate based autopolymerized resin, bis-acrylic composite based autopolymerized resin and a light polymerized resin). When the mean flexural strength of three provisional crown materials ( Table 1) was consi-dered the methyl methacrylate based autopolymerized resin showed the highest flexural strength followed by the light polymerized resin and bis-acrylic composite based autopolymerized resin showed least flexural strength. When repeated measure ANOVA ( Table 2) was applied to mean flexural strength values of 3 materials at three different storage intervals in artificial saliva, a significant F value was observed (F=7.667; P=.000) verifying change in the flexural strength with respect to individual materials.

**Table 1:**
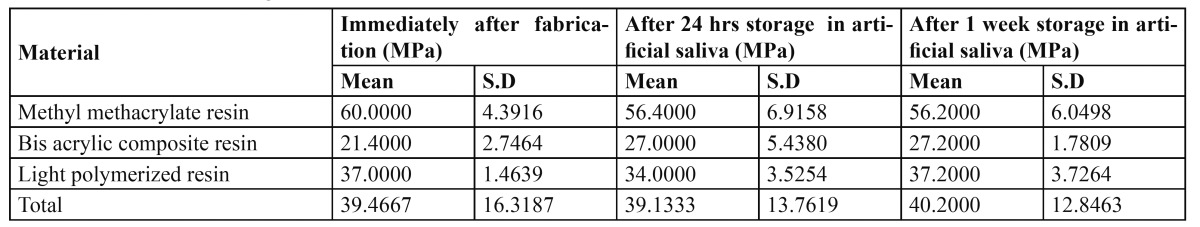
Mean flexural strength values of different materials at three different time intervals.

**Table 2:**
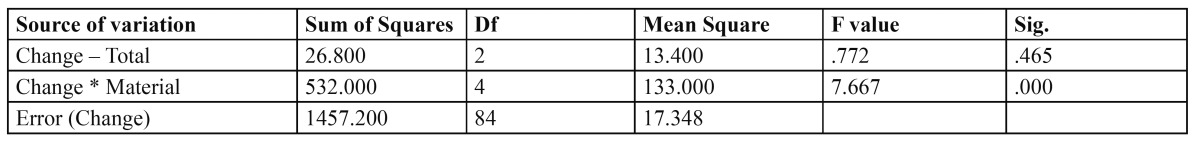
Tabulated results of repeated measure ANOVA for mean flexural strength values of different materials at three different time intervals.

Methyl methacrylate based resin reduced in flexural strength significantly after 24 hours storage in artificial saliva and remained constant to the 7 days storage time. However, bis-acrylic composite resin observed an increase in its flexural strength after 24 hours storage in artificial saliva and did not show significant change after 7 days, where as light polymerized resin decreased in flexural strength after 24 hours storage in artificial saliva and thereafter an increase in the flexural strength values after 7 days.

When the mean elastic moduli of three provisional crown materials ( Table 3) was considered methyl methacrylate based resin showed the highest elastic moduli followed by light polymerized resin and bis-acrylic composite based resin showed the least elastic moduli of the three. When repeated measure ANOVA ( Table 4) was applied to mean elastic moduli values of the 3 materials at three different storage intervals in artificial saliva a non-significant F value was observed (F=7.667; P=.000), verifying no significant change in the elastic moduli with respect to the individual materials after storage in artificial saliva.

**Table 3:**
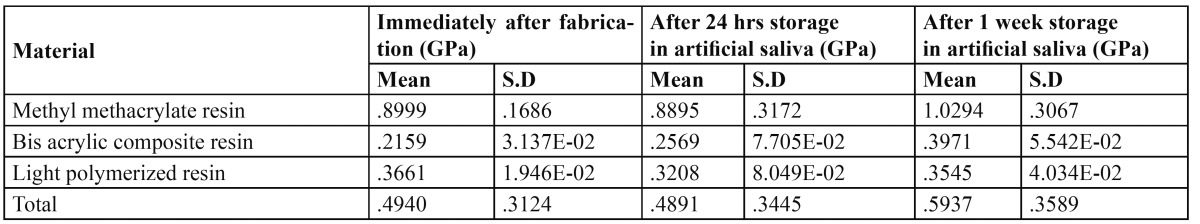
Mean elastic moduli values of different materials at three different time intervals.

**Table 4:**
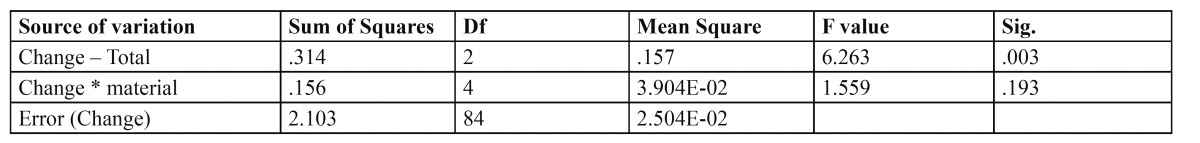
Results of repeated measure ANOVA for mean elastic moduli values of different materials at three different time intervals.

## Discussion

Contemporary provisional restorations have traditionally been resin based. These resin based restorative materials offer advantages like esthetics and accuracy of fit. Yet durability in service with regard to their mechanical properties remains an area that has been a subject of much debate. The arrival of newer composite resin based provisional restorative materials to an area dominated by polymethyl methacrylate has lent a freedom of choice to the practitioner. It has yet added to the conundrum of doubt, regarding their performance in vivo. These variations with regard to their mechanical parameters are likely to be due to the differences in their method of polymerization, filler composition and monomer type. While polymethyl methacrylates happen to be the time tested restorative material with a legacy of service, the bis-acrylic composites and urethane dimethacrylates have undergone several modifications as provisional restorative material.

Bis-acrylic composite based provisional restorative materials are gaining in popularity, in part because of their cartridge delivery system. This dispensary method is not only convenient but also may allow for a more accurate and consistent mix (7) and thereby improving its physical and mechanical properties. Hence this study designated polymethyl methacrylate as the standard against which the bis-acrylic composite based restorative material and the light cured urethane dimethacrylate restorative material were assessed.

The parameters given due consideration in this study were flexural strength and elastic modulus as these attain significance from a clinical standpoint. Flexural strength, also known as transverse strength, is a measurement of the strength of a bar (supported at each end) under a static load. The flexural strength test is a combination of tensile and compressive strength tests and includes elements of proportional limit and elastic modulus measurements (1). Elastic modulus (E) describes the relative stiffness or rigidity of a material. With the data obtained from this study on measuring the flexural strength and elastic moduli of provisional restorative materials, it was observed that methacrylate based autopolymerized resin showed superior flexural strength and elastic moduli in comparison to both light polymerized and bis-acrylic composite based autopolymerized resin after fabrication and after storage, while the bis-acrylic composite based resin exhibited the least flexural strength and elastic moduli amongst the three.

This increased flexural strength of the polymethyl methacrylate resins compared to the autopolymerized bis-acrylic composites and light polymerized resins concurs with the study done by Osman et al. (8) who tested five autopolymerizing provisional resin materials under conditions that related the stresses acting on them to those acting on a fixed partial denture. The highest values for fracture resistance in that study were displayed by poly (ethyl methacrylate) material (Snap™). In decreasing order, the fracture resistance of the other materials was as follows: the poly (methyl methacrylate) materials, Caulk™ temporary bridge resin and G-C Unifast™ temporary resin; the composite material, Protemp™; and the epimine material, Scutan™ (8).

Similar results were obtained by Scherrer et al (9) and Haselton et al (1). However Koumjian et al, (4) concluded that even though no data were available to compare the type of resin matrix or filler content of those bis-acrylic composite materials, it was evident that the difference in flexural strength performance was material specific. Direct comparison to other studies was not possible due to differences in materials, methodology, and specimen configuration. A review of the limited research on flexural strength of provisional materials also showed this property to be material specific. (4,8,10) Monomers associated with different provisional materials impart different characteristics such as exothermic heat of reaction, polymerization shrinkage, and strength. The results of the study by Haselton et al (1) demonstrated that flexural strengths vary greatly among provisional materials and that there seemed to be no correlation between flexural strength and type of provisional dental resin (1). It was also observed in the study by Haselton et al (1) that there is a significant increase in the flexural strength of Protemp™ 3 Garant compared to its predecessor Protemp™ Garant (bis-acrylic composites). This is due to the modifications in Protemp™ 3 Garant that include a newly developed monomer system, not with the rigid intermediate chain characteristic of some bis-acrylic homologues, but with a somewhat flexible chain that allows a balance between high mechanical strength and limited elasticity of the composite material (1). The direct comparison between the hand-mixed Protemp™ II bis-acryl composite provisional crown material (used in this study) and its automixed version (Protemp™ Garant) showed no difference in the Weibull distribution, thus indicating that the distribution of flaws inside the set material is identical and independent of the type of mixing (9). This could explain the decreased flexural strength and elastic moduli of Protemp™ II bis-acrylic composite provisional crown material compared to the DPI™ polymethyl methacrylate provisional crown material.

Poly (methyl methacrylate) absorbs small amounts of water when placed in an aqueous environment. The water molecules penetrate the Poly (methyl methacrylate) mass, and occupy positions between polymer chains and the affected polymer chains are forced apart. The decrease in flexural strength of the methacrylate based autopolymerized resin after immersion could be because the water molecules interfere with the entanglement of polymer chains, and thereby act as plasticizers (8,11).

Protemp™ II is a bis-acryl resin containing bifunctional methacrylate (70%), silicone dioxide as filler (25%), vinyl copolymers (4%), inorganic fillers (56%) and bifunctional esters (40%). It is hydrophobic (12), ensuring minimal water uptake and thus reducing the plasticizer action. In addition, vinyl copolymers are included to increase the flexural strength. Bis-acryls have a rigid central structure that reduces the dissolution of the res-in-filler particles during its immersion in saliva (13). This, along with the continued polymerization of the composite resin material could have led to the increase in flexural strength of the Protemp™ II material after storing in artificial saliva for 24 hours. This finding regarding Protemp™ II concurs with the study done by Koumjian et al (4) who tested seven resins namely, Cold pac™, Duralay™, Protemp™, Snap™, Triad™, Trim™, and Trukit™ for fracture resistance and the effects of water absorption and repair. His study showed that Triad™, Protemp™, Snap™ and Trim™ resins showed significant increases in transverse strength after seven days wet storage (4).

The new monomer system developed by 3M ESPE for the successors of Protemp™ II bis acry composite mate-rial like Protemp™ 3 Garant and Protemp™ 4 Garant offers outstanding mechanical strength and high resistance to fracture without the brittleness associated with composites (14).

The Revotek™ LC material contains urethane dimethacrylate (45-50%), and crystalline silica powder (10-15%) as filler. Less filler particles are found in interim composites (15-35%) by weight compared to normal composites (85%) by weight (15). This could be the reason for reduced strength of the material. The glass fillers are slowly leached out in the presence of saliva, thus explaining reduction in mechanical properties of the interim composite after storage (12).

Interestingly, Revotek™ LC shows a significant increase in flexural strength 24 hours to 7 days of storage. The light cure nature may have allowed more continual cross linking to take place between 24 hours and 7 days of storage and contribute to the significant increase in flexural strength countering the degradation effect from soaking.

Kamble et al (14) has shown that Glass and Polyethylene fibers improved the fracture toughness of the specimens compared to the unreinforced methyl methacrylate and bis-acrylic composite resin. This shows that, use of fibers is an effective method to increase the mechanical properties of the provisional restorative resins (14).

It is important to note that although methyl methacrylate based material responded with the best mechanical properties in the experiment, it does not necessarily mean that it is the best interim fixed prosthetic material. With many choices of materials available to use as interim restorations, it is important for clinicians to make their selection based upon the clinical needs for each situation. As part of these considerations, clinicians must understand and factor in the physical properties, handling characteristics, patient response to the appearance of the interim restoration, durability of the restoration, and the material cost in deciding which material to use. No one material meets all the requirements for provisional restorations. Selection of provisional materials should be made based upon a case-by-case evaluation for any given patient (2).

Within limitations of this study the following conclusions were drawn:

1. The methacrylate based autopolymerizing resin (DPI™) showed the highest flexural strength and elastic moduli after fabrication, after storage in artificial saliva and testing the specimens at intervals of 24 hours and 7 days. The bis-acrylic composite resin (Protemp™ II) showed the least flexural strength and elastic moduli among the three.

2. Flexural strength of the methyl methacrylate based materials reduced significantly after storing for 24 hours in artificial saliva and remained constant up to 7 days of storage.

3. Flexural strength of the bis-acrylic composite resin significantly increased after storing for 24 hours and did not show significant change after storing for 7 days in artificial saliva.

4. Flexural strength of the light polymerized resin (Revotek™ LC) decreased after storing for 24 hours and significantly increased after storing for 7 days in artificial saliva.

5. There was no significant change in the elastic moduli with respect to the individual materials after storing in artificial saliva for 24 hours and 7 days.
